# Unveiling the hidden AP-1: revealing the crucial role of AP-1 in ccRCC at single-cell resolution

**DOI:** 10.1186/s12943-023-01913-9

**Published:** 2023-12-19

**Authors:** Jie Zheng, Fengling Liu, Cheng Su

**Affiliations:** 1 Department of Urology, The First Affiliated Hospital of Guangxi Medical University, Nanning, Guangxi China; 2 Department of Pediatric Surgery, The First Affiliated Hospital of Guangxi Medical University, Nanning, Guangxi China; 3 Department of Hematology, The First Affiliated Hospital of Guangxi Medical University, Nanning, Guangxi China; 4 Center for Genomic and Personalized Medicine, Guangxi key Laboratory for Genomic and Personalized Medicine, Guangxi Collaborative Innovation Center for Genomic and Personalized Medicine, Guangxi Medical University, Nanning, Guangxi China

**Keywords:** AP-1, Clear cell renal cell carcinoma, scATAC, scRNA, Spatial transcriptomics

## Abstract

Clear cell renal cell carcinoma (ccRCC), as the most common histological subtype of kidney cancer, has been reported to originate primarily from proximal tubule (PT) cells in the kidney. However, the current research on its associated molecular mechanisms remains relatively limited. In our study, we analyzed multiple single-cell multi-omics datasets obtained from various research teams, revealing the significant role of the activator protein 1 (AP-1) in ccRCC tumorigenesis. The motif activity analysis of transcription factors (TFs) showed a predominant activation of AP-1 in ccRCC cancer cells compared to PT cells. Furthermore, our findings at single-cell resolution revealed a notable absence of AP-1 expression in PT cells when compared to ccRCC cancer cells. In bulk-RNA of discovery cohort, no differential expression of AP-1 was detected in normal kidney and ccRCC samples, which may be attributed to confounding effects in bulk-RNA sequencing. Meanwhile, spatial transcriptomics analysis demonstrated a broader expression range of the AP-1 compared to the ccRCC marker CA9. Moreover, we observed chromatin accessibility of the AP-1 in various cell-types, including PT cells, suggesting that the transcriptional expression of AP-1 in PT cells may be influenced by subsequent transcriptional modifications, reflecting the complex regulatory mechanism of AP-1 transcription. These findings provide important insights for a deeper understanding of the function and regulatory mechanisms of AP-1 in ccRCC, thereby establishing a theoretical foundation for future clinical research and the development of treatment strategies.

**Supplementary Information:**

The online version contains supplementary material available at 10.1186/s12943-023-01913-9.

Clear cell renal cell carcinoma (ccRCC) stands as the prevailing and aggressive histological subtype of kidney cancer, exhibiting a continuous rise in global incidence and mortality rates each year [1]. The inconspicuous clinical presentation and invasive nature of ccRCC play a substantial role in the occurrence of metastasis at the time of primary diagnosis [2]. Despite the progress made in therapeutic interventions for metastatic ccRCC, patients in this particular category face unfavorable prognoses, with an overall 5-year survival rate of merely 10% [3]. Currently, numerous reports suggest that proximal tubule cells are the primary source of ccRCC cancer cells [4, 5]. However, our current understanding of the molecular mechanisms underlying ccRCC tumorigenesis remains relatively limited. It is imperative to comprehensively elucidate the underlying mechanisms governing ccRCC tumorigenesis, as this will establish a fundamental theoretical basis for the development of effective treatment strategies.

The AP-1 (activator protein 1) transcription factor is a dimeric complex composed of members from the JUN (c-Jun, JunB, and JunD), FOS (c-Fos, FosB, Fra-1, and Fra-2), ATF, and MAF protein families. AP-1 proteins form dimeric complex through a leucine-zipper motif, and the activity of the AP-1 complex is influenced by the composition of dimers as well as the cellular and genetic context. The specific dimer composition determines the downstream genes that are regulated [6]. AP-1 participates in a wide range of physiological and pathological processes. Particularly in tumorigenesis and progression, numerous studies have documented its significant role [6, 7]. Despite extensive research on AP-1 in various cancer types, our understanding of its specific involvement in ccRCC, particularly in elucidating the ccRCC tumorigenesis, remains limited. With the advancements in single-cell genomics, we can now analyze the occurrence of specific molecular events at a high resolution [8, 9], which greatly facilitates the elucidation of the role of the AP-1 in ccRCC.

In this study, we aimed to elucidate the role of AP-1 in ccRCC tumorigenesis by integrating high-resolution single-cell multi-omics data, thereby providing a theoretical foundation for clinical investigations and the development of treatment strategies.

## The crucial role of AP-1 in ccRCC tumorigenesis

The origin of ccRCC is intricately connected to specific cell types and molecular events occurring within the kidney. Extensive research suggests that the majority of ccRCC cases stem from PT cells, wherein mutations and abnormal activation of multiple key genes contribute to the atypical proliferation and transformation of tumor cells [4, 5]. Among these genetic alterations, the most prevalent change involves the accumulation of HIF transcription factors due to VHL mutations, subsequently triggering a cascade of downstream alterations in target genes [10]. The widespread impact of TFs in most biological events makes them pivotal players. In this context, single-cell assay for transposase-accessible chromatin using sequencing (scATAC-seq) emerges as a valuable technique, offering unique capabilities to investigate the binding and activity of TFs.

In this study, scATAC data including three ccRCC tumor samples and five normal kidney samples were collected as discovery cohort to investigate the molecular characteristics of ccRCC at the DNA level (source of data see Table S1). After implementing quality control procedures, we retained a total of 63,489 cells for further analysis (Quality control criteria described in the supplementary methods). By utilizing established molecular markers, we classified these cells into 14 distinct subgroups (Figure S1A) [9, 11]. The gene activities for these markers were depicted in Figure S1B. Gene activity was determined by assessing the accessibility of chromatin peaks, encompassing a region of the gene body and the upstream 2 kb region of the transcription start site. The chromatin open peaks of CA9 and SLC34A1 serve as specific markers for ccRCC cancer cells and PT cells, respectively, illustrating their discriminatory significance (Figure S1C).

**Fig. 1:**
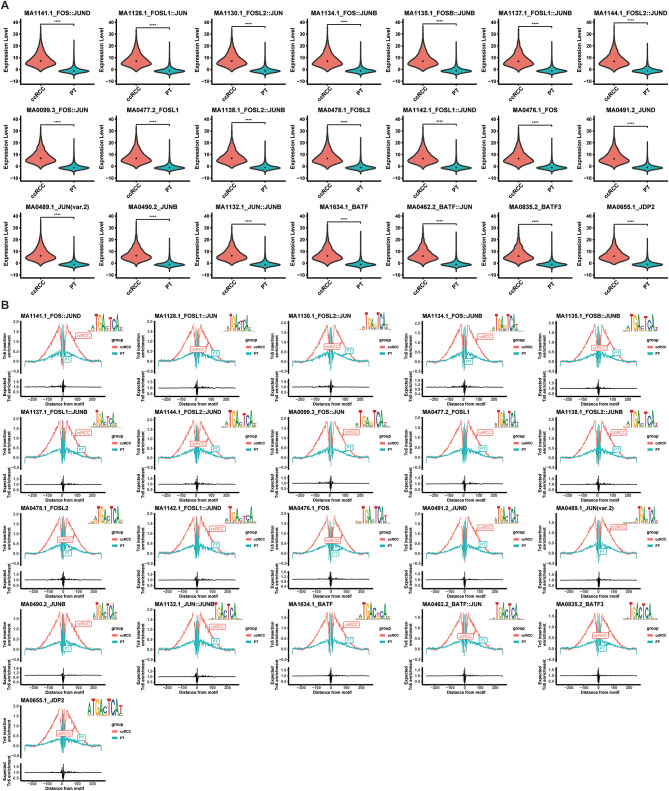
Motif activity and footprint analysis of AP-1 in ccRCC cancer cells and PT cells. (**A**) Motif activity comparison of AP-1 between ccRCC cancer cells and PT cells. The red represented ccRCC cancer cells, the blue-green represented PT cells. (**B**) Footprint analysis of AP-1 motifs in ccRCC cancer cells and PT cells. The red represented ccRCC cancer cells, the blue-green represented PT cells. The footprint analysis is for computing the normalized Tn5 insertion frequency for each position surrounding AP-1 motif instances. The logo in the upper right corner represents motif logo, which provides a visual summary of the relative frequency of each nucleotide at each position within the motif. ccRCC, clear cell renal cell carcinoma; PT, proximal tubule

Our subsequent analysis focuses primarily on examining the activity of transcription factors, which was reflected by the accessibility of transcription factor binding sites (TFs-motif activity). Through the analysis of differential TFs-motif activity between ccRCC cancer cells and PT cells, we were surprised to discover that among the top 22 differentially active transcription factors, 21 of them belonged to the AP-1 transcription factor family (Fig. 1A, Table S2, avg_diff > 7, *p* < 0.05). More specifically, these differentially active AP-1 transcription factors family predominantly comprised individual AP-1 proteins from the FOS and JUN families, as well as the dimers formed by them. This finding highlights the dominant activation of the AP-1 transcription factor family in ccRCC cancer cells compared to PT cells. To further delve into this phenomenon, we employed TFs-footprinting analysis, which revealed a robust binding affinity of the AP-1 transcription factor family to DNA in ccRCC cancer cells compared to PT cells (Fig. 1B). These findings unequivocally underscore the critical role played by the AP-1 in the ccRCC tumorigenesis.

To validate our findings, we conducted a validation cohort analysis using a large-scale scATAC dataset comprising 18 ccRCC tumor samples (source of data see Table S1). After applying appropriate filters (as described in the supplementary methods), the dataset comprised 41,585 cells, which were subjected to subsequent analysis. Utilizing classical markers, we categorized these cells into 11 distinct subgroups (Figure S2A). The gene activity profiles corresponding to each subgroup are visually depicted in Figure S2B. Consistent with the findings of the discovery dataset, we observed a robust binding of the AP-1 transcription factor family to DNA within ccRCC cancer cells, indicating its abnormal activation in this specific context (Figure S2C).

## Unveiling the masked AP-1 at single-cell resolution

In our previous analysis utilizing scATAC data, we discovered significant disparities in the motif activity of the AP-1 between ccRCC cancer cells and PT cells. However, it is important to acknowledge that the expression of AP-1 also partially reflects their activity. Consequently, to delve further into the transcriptional expression of AP-1 family members, we collected scRNA data encompassing three ccRCC tumor samples and three normal kidney samples to investigate ccRCC’s molecular characteristics at the transcriptome level (source of data see Table S1). Following rigorous quality control (as described in the supplementary methods), we retained 50,604 cells for subsequent analysis. These cells were then classified into 16 subgroups based on established molecular markers (Figure S3A). The gene expression associated with these markers is illustrated in Figure S3B.

**Fig. 2:**
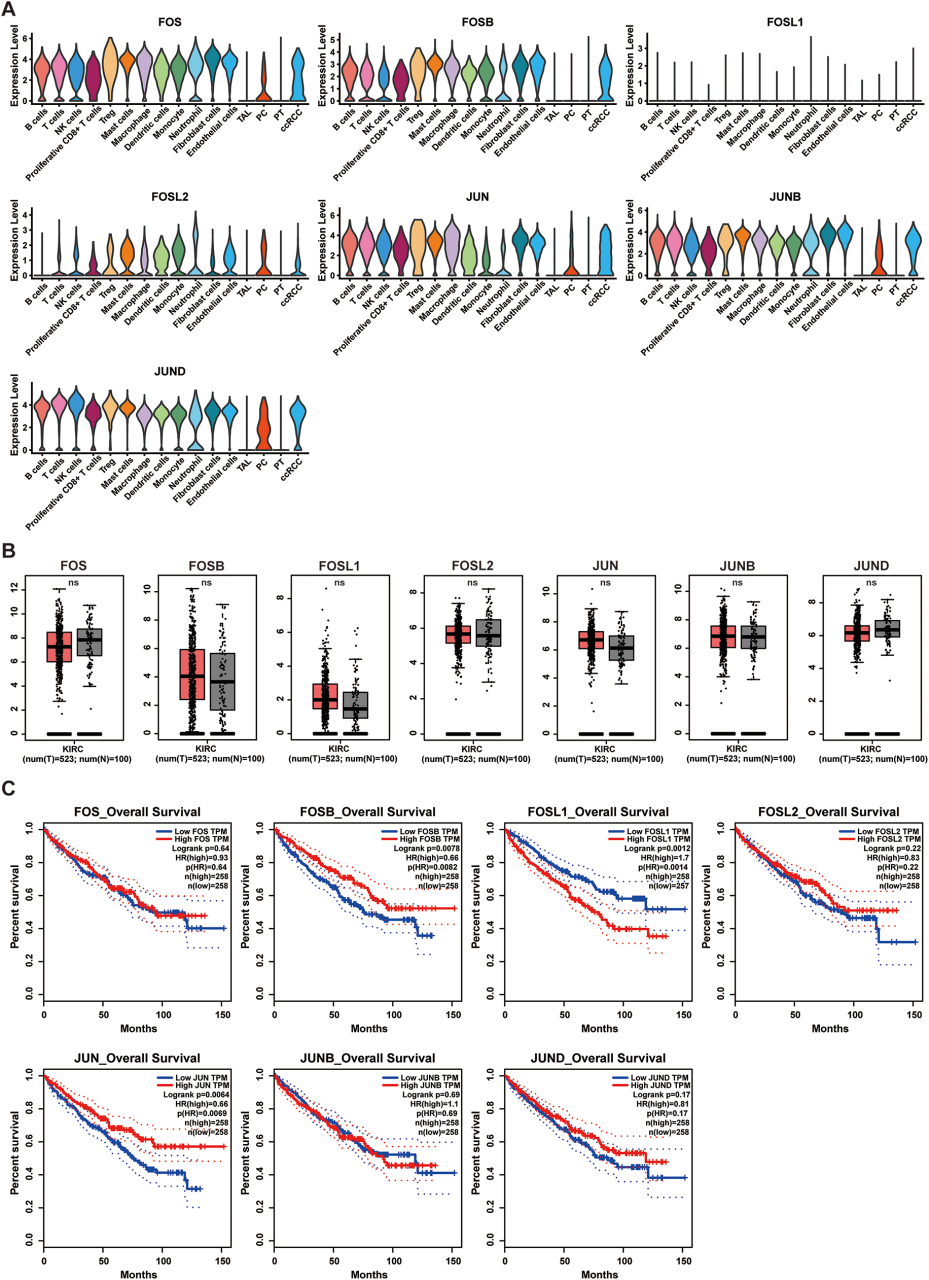
Unveiling masked AP-1 expression at single-cell resolution. (**A**) Expression levels of FOS and JUN family genes in ccRCC cells based on scRNA-seq data. ccRCC, clear cell renal cell carcinoma; PT, proximal tubule; PC, principal cell; TAL, thick ascending limb. (**B**) Comparison of FOS and JUN family gene expression between ccRCC and normal kidney samples based on bulk-RNA data. The red represented ccRCC tumor samples, the gray represented normal kidney samples. (**C**) Survival analysis of FOS and JUN family genes in ccRCC based on bulk-RNA data

At the single-cell resolution, we observed a deletion of gene expression for c-FOS, FOSB, and FOSL2 from the FOS family, as well as c-JUN, JUNB, and JUND genes from the JUN family, in PT cells. In contrast, ccRCC cancer cells exhibited robust expression of these genes (Fig. 2A). Meanwhile, these genes are also expressed in various cell types, including immune cells and stromal cells, apart from ccRCC cancer cells (Fig. 2A). In relation to other genes within the AP-1 family, specifically those belonging to the ATF and Maf families, the majority of them demonstrated no expression in any type of ccRCC cells. However, *ATF3*, *ATF4*, *MAF*, and *MAFF* were exceptions, as they were found to be expressed in certain cell types and exhibited significantly higher expression levels compared to PT in ccRCC cancer cells (Figure S3C). This observation was further validated in an additional scRNA dataset comprising 18 ccRCC samples and 112,820 cells (Figure S4A-4 C).

Through our analysis at the single-cell level, we have uncovered noteworthy disparities in the expression of AP-1 between PT cells and ccRCC cancer cells. This finding underscores the pivotal role of AP-1 in the ccRCC tumorigenesis. Nevertheless, it is important to note that genes associated with AP-1 are not exclusively expressed in ccRCC cancer cells but are also present in other most cell types. Hence, the expression of AP-1 might be obscured when employing bulk-RNA sequencing to compare differentially expressed genes between normal kidney and ccRCC tissues. The analysis of the TCGA dataset indeed demonstrated that there were no statistically significant differences in gene expression within the FOS and JUN families when comparing ccRCC and normal kidney samples (Fig. 2B). In our validation cohort, we observed increased expression of *JUN* and *JUNB* in ccRCC tumor tissues, whereas *FOS* and *JUND* exhibited higher expression in normal kidney tissues. Consistent with the TCGA dataset, *FOSB*, *FOSL1*, and *FOSL2* demonstrated no significant expression differences between ccRCC tumor and normal kidney tissues (Figure S5A). Furthermore, in the survival analysis of TCGA dataset, with the exception of FOSL1, which exhibited low expression and a correlation with a poor prognosis in ccRCC, other genes in the FOS and JUN families demonstrated either a favorable prognosis or no significant implications for survival (Fig. 2C, *p* *<* 0.05). These findings offer valuable insights and cautionary considerations, particularly when evaluating gene function through differential and survival analyses based on bulk-RNA sequencing data. Our study on AP-1 demonstrates the evident impact of non-tumor cell contamination in bulk-RNA sequencing.

Moreover, we expanded our investigation to include spatial transcriptomics, which provided supplementary insights into the transcriptional expression of genes in the JUN and FOS families. Our analysis revealed that FOS and JUN family genes displayed a broader range of expression compared to the ccRCC marker CA9, corroborating the findings from single-cell transcriptomics (Figure S5B). Meanwhile, we also leveraged scATAC data to assess the chromatin accessibility of genes associated with the JUN and FOS families. Interestingly, our study revealed a noteworthy observation: the JUN and FOS family gene displayed a certain level of chromatin accessibility in PT cells (Figure S6). This observation suggests that the transcriptional expression of AP-1 in PT cells is influenced by subsequent transcriptional modifications, underscoring the intricate regulatory mechanisms that govern AP-1 transcription.

Prominent discrepancies in the transcription factors activity and expression of AP-1 have been noted between PT and ccRCC cancer cells, underscoring their crucial involvement in ccRCC tumorigenesis. It is worth highlighting that the target genes under direct regulation by AP-1 play an active role in relevant biological processes. To explore this further, we employed the pySCENIC tool for the initial screening of the co-expression network of AP-1-regulated target genes, followed by an investigation of the upregulated genes in ccRCC cancer cells in comparison to PT cells (Figure S7A, Table S3). Our investigation unveiled a remarkable enrichment of target genes related to AP-1 in various pathways intricately associated with tumorigenesis and tumor progression, including TNF, hypoxia, P53, EMT, apoptosis, MAPK, focal adhesion, and WNT signaling pathways (Figure S7B-S7D). These findings further reinforce the substantial involvement of AP-1 in the tumorigenesis and progression of ccRCC.

This study has several limitations that should be acknowledged. Firstly, all analyses conducted in this study relied solely on bioinformatics findings, necessitating further experimental validation. Secondly, the inclusion of scRNA and scATAC data from normal kidneys that are matched to the ccRCC data in the validation cohort would enhance the credibility of our findings.

## Conclusion

In conclusion, we employed single-cell multi-omics analysis to gain initial insights into the significant activation of the AP-1 in ccRCC cancer cells compared to PT cells. This finding emphasizes the pivotal role of AP-1 in the tumorigenesis of ccRCC. Furthermore, the absence of AP-1 expression in PT cells, as detected at the single-cell level, highlights the importance of accounting for the confounding effects of cell heterogeneity in bulk-RNA sequencing studies. Additionally, our investigation of chromatin accessibility associated with AP-1 suggests that the transcriptional expression of AP-1 in PT cells may be subject to subsequent transcriptional modifications, highlighting the intricate regulatory mechanisms governing AP-1 transcription. These findings provide valuable insights for a deeper comprehension of the function and regulatory mechanisms of AP-1 in ccRCC, thereby establishing a theoretical foundation for future clinical research and the development of therapeutic strategies.

### Electronic supplementary material

Below is the link to the electronic supplementary material.

**Supplementary Material 1: Figure S1**. Single-cell epigenome profiles of ccRCC and normal kidney based on discovery cohort. **Figure S2**. Single-cell epigenome profiles of ccRCC based on validation cohort. **Figure S3**. Single-cell transcriptome profiles of ccRCC and normal kidney based on discovery cohort. **Figure S4**. Single-cell transcriptome profiles of ccRCC and normal kidney based on validation cohort. **Figure S5**. Comparing FOS and JUN family gene expression between ccRCC and normal kidney samples and the spatial transcriptome expression of AP-1. **Figure S6**. Chromatin accessibility of AP-1 in scATAC-seq data. **Figure S7**. Functional enrichment analysis based on AP-1 target genes. **Supplementary Methods**. **Supplementary Information References**

**Supplementary Material 2: Table S1**. Data source and Quality control in this study

**Supplementary Material 3: Table S2**. Differential motif activities in cancer cells vs. proximal tubule cells

**Supplementary Material 4: Table S3**. Target gene of AP-1

## Data Availability

The data sources utilized in this study are summarized in Table S1 and all data is publicly downloadable.

## Abbreviations

AP-1: Activator protein 1
ccRCC: Clear cell renal cell carcinoma
GEO: Gene Expression Omnibus
OS: Overall survival
PT: Proximal tubule
scATAC-seq: Single-cell assay for transposase-accessible chromatin using sequencing
scRNA-seq: Single-cell RNA sequencing
stRNA-seq: Spatial transcriptomics sequencing
TCGA: The Cancer Genome Atlas
TFs: Transcription factors
