# Supplementary material for: Unveiling the hidden AP-1: revealing the crucial role of AP-1 in ccRCC at single-cell resolution
Source: Mol Cancer. 2023 Dec 19;22:209. doi: 10.1186/s12943-023-01913-9 (PMC10731896; doi:10.1186/s12943-023-01913-9)
Supplement: Supplementary file 1 — Supplementary Material 1: Figure S1. Single-cell epigenome profiles of ccRCC and normal kidney based on discovery cohort. Figure S2. Single-cell epigenome profiles of ccRCC based on validation cohort. Figure S3. Single-cell transcriptome profiles of ccRCC and normal kidney based on discovery cohort. Figure S4. Single-cell transcriptome profiles of ccRCC and normal kidney based on validation cohort. Figure S5. Comparing FOS and JUN family gene expression between ccRCC and normal kidney samples and the spatial transcriptome expression of AP-1. Figure S6. Chromatin accessibility of AP-1 in scATAC-seq data. Figure S7. Functional enrichment analysis based on AP-1 target genes. Supplementary Methods. Supplementary Information References [file 12943_2023_1913_MOESM1_ESM.docx]

**Supplementary Figure 1**

**
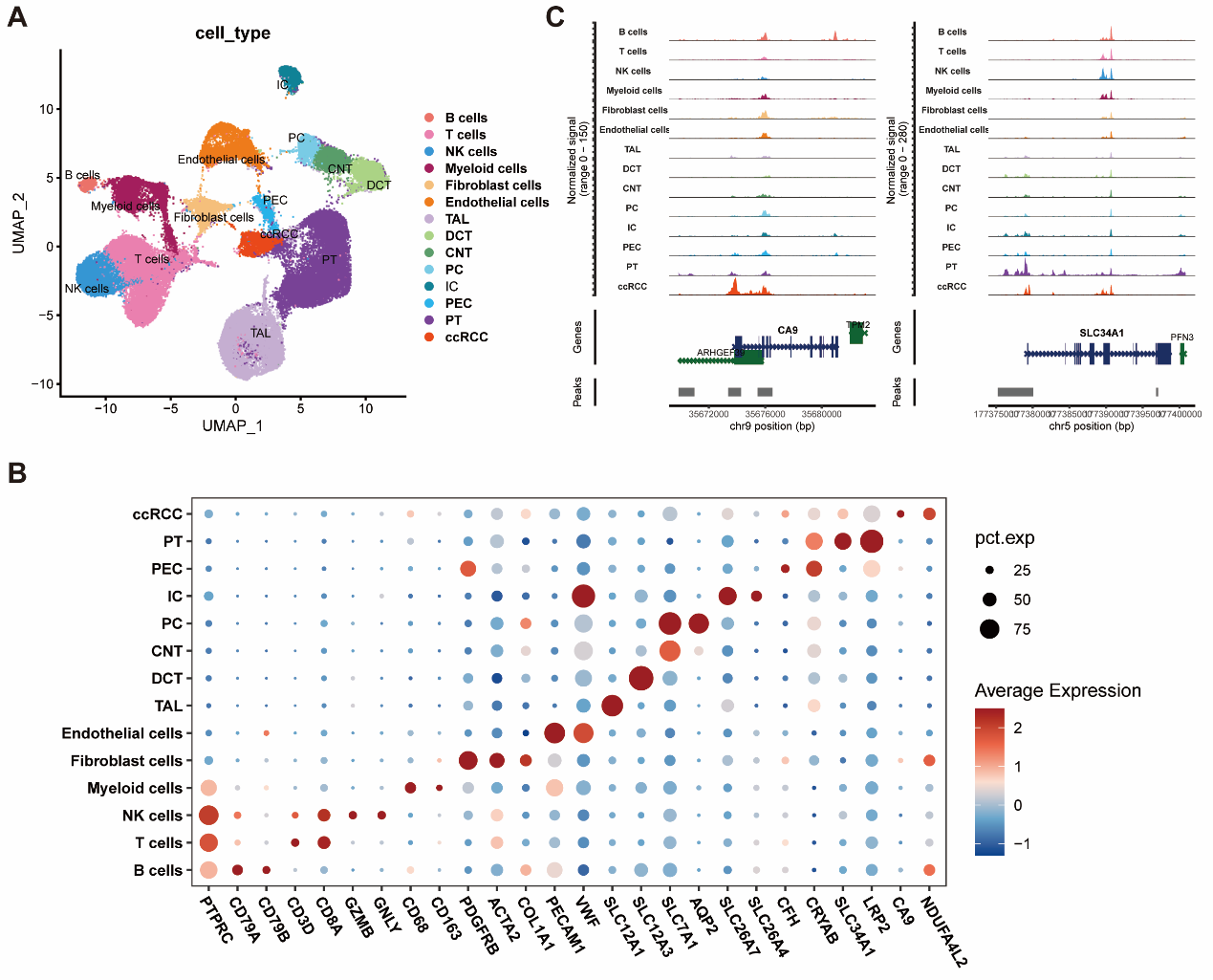
**

**Figure S1. Single-cell epigenome profiles of ccRCC and normal kidney based on discovery cohort.**

1. UMAP embedding of cells derived from scATAC-seq data, including 3 ccRCC tissues and 5 normal kidney tissues. (B) Dot plot illustrating the gene activity of marker genes for each cell type in scATAC-seq data. Gene activity was determined by assessing the accessibility of chromatin peaks, encompassing a region of the gene body and the upstream 2kb region of the transcription start site. (C) Chromatin accessibility of CA9 and SLC34A1 gene in scATAC-seq data. ccRCC, clear cell renal cell carcinoma; CNT, connecting tubule; DCT, distal convoluted tubule; IC, Intercalated cells; PC, principal cell; PEC, parietal epithelial cells; PT, proximal tubule; TAL, thick ascending limb.

**Supplementary Figure 2**


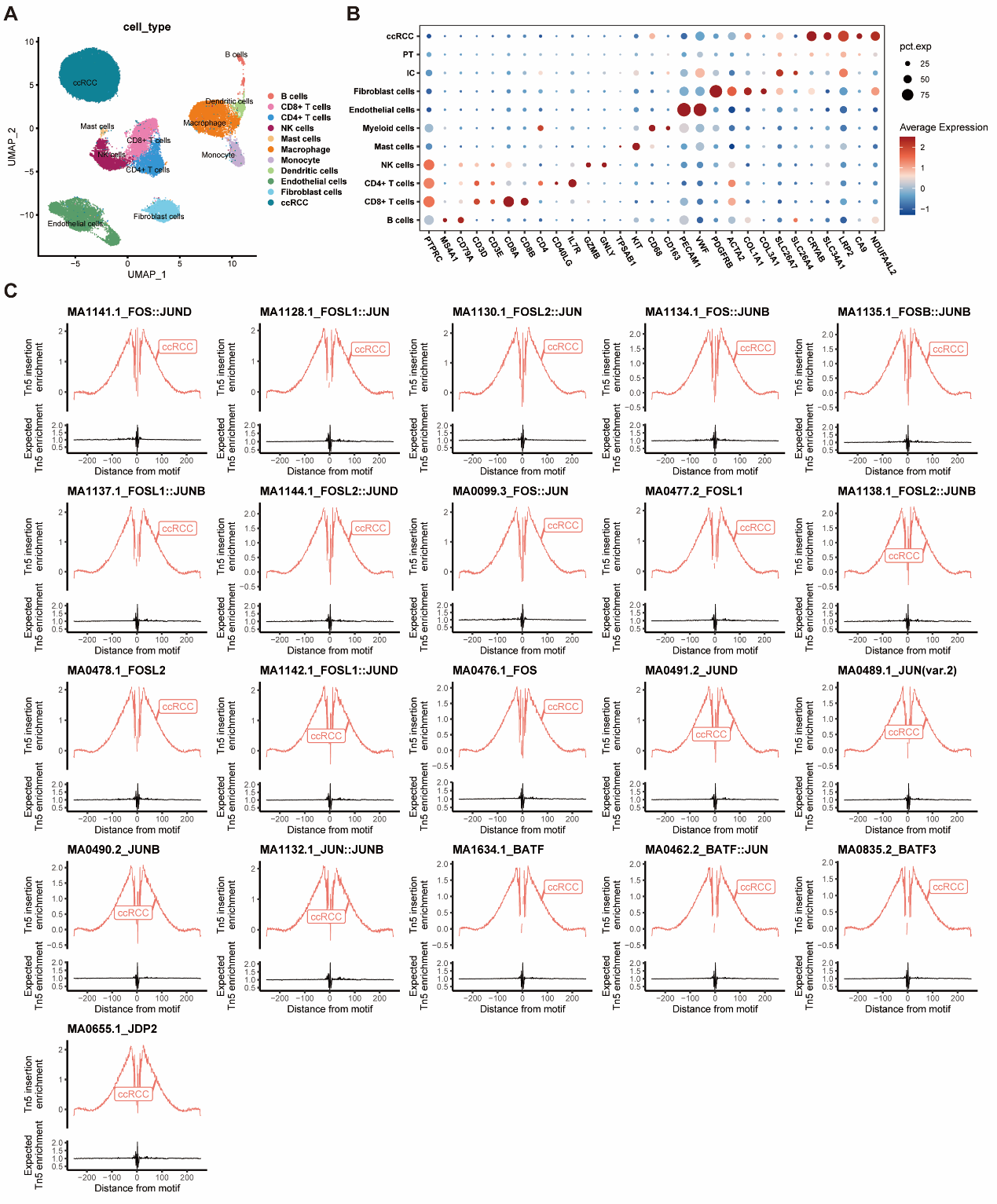


**Figure S2. Single-cell epigenome profiles of ccRCC based on validation cohort.**

1. UMAP embedding of cells derived from scATAC-seq data, including 18 ccRCC samples. (B) Dot plot illustrating the gene activity of marker genes for each cell type in scATAC-seq data. (C) Footprint analysis of AP-1 motifs in ccRCC cancer cells.

**Supplementary Figure 3**


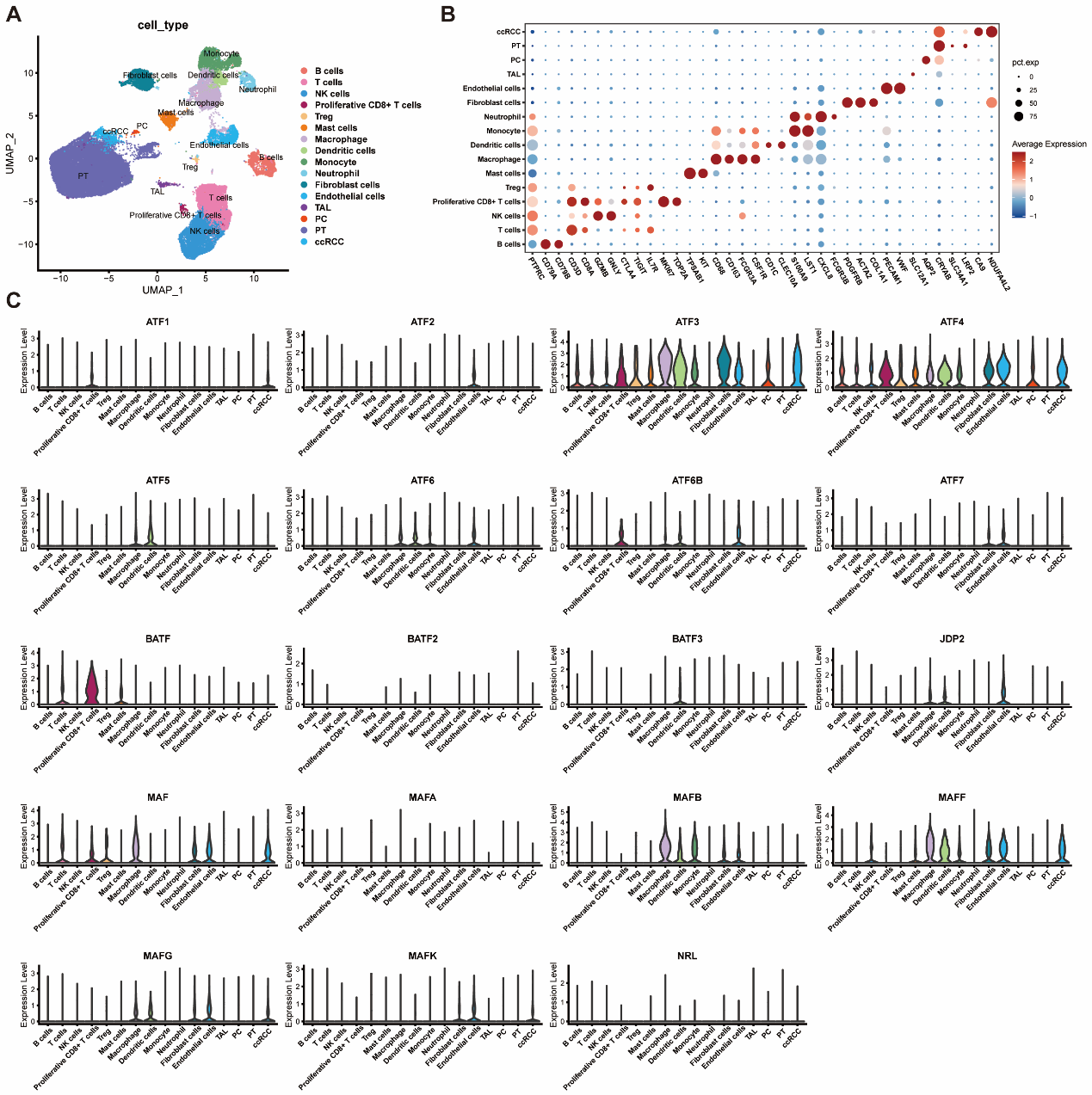


**Figure S3. Single-cell transcriptome profiles of ccRCC and normal kidney based on discovery cohort.**

1. UMAP embedding of cells derived from scRNA-seq data, including 3 ccRCC tissues and 3 normal kidney tissues. (B) Dot plot illustrating the expression patterns of marker genes for each cell type in scRNA-seq data. The size of the dots represents the percentage of gene expression within the cell subgroups, while the color indicates the intensity of expression. (C) Expression levels of ATF and MAF family genes in ccRCC cells based on scRNA-seq data. ccRCC, clear cell renal cell carcinoma; PT, proximal tubule; PC, principal cell; TAL, thick ascending limb.

**Supplementary Figure 4**

**
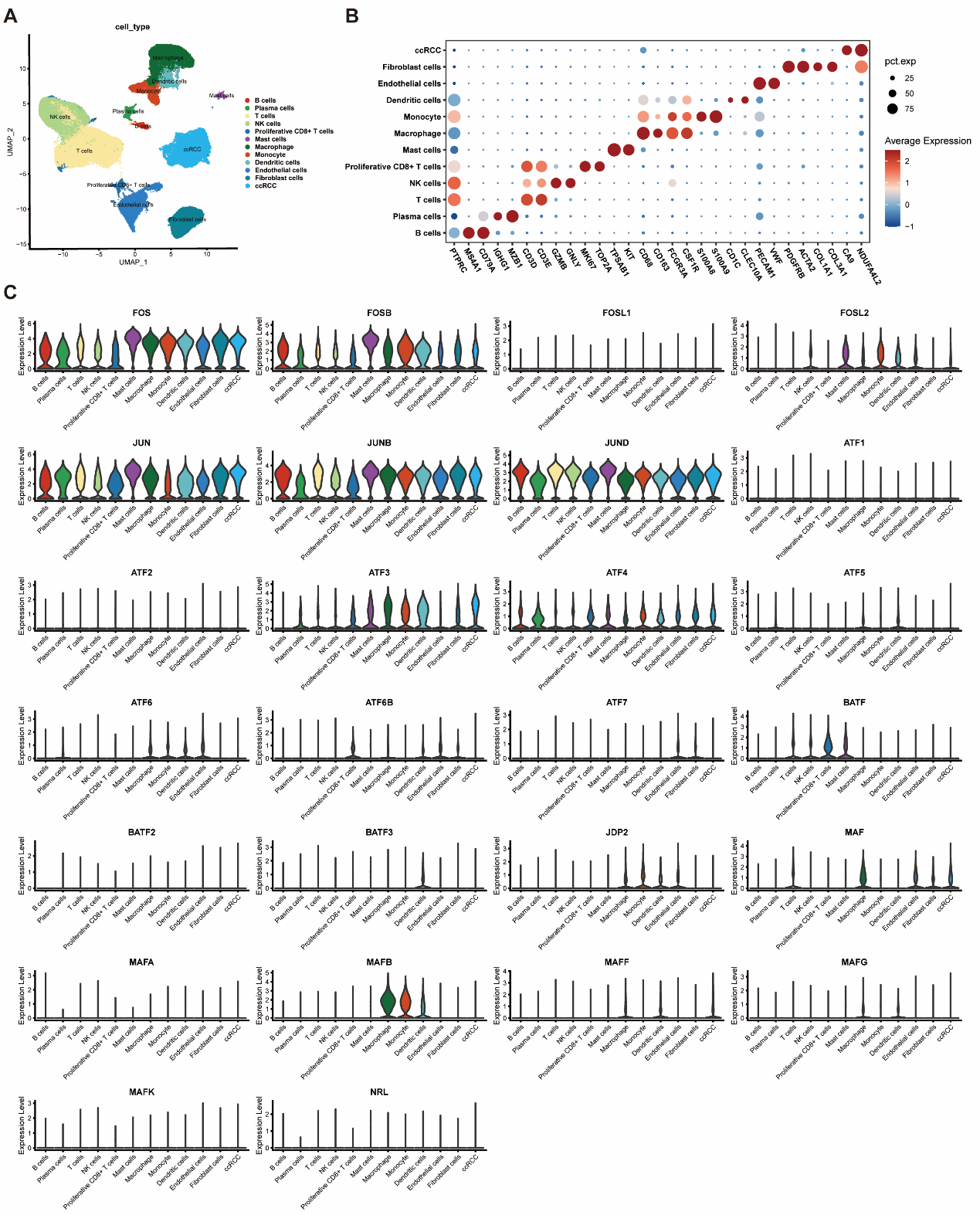
**

**Figure S4. Single-cell transcriptome profiles of ccRCC and normal kidney based on validation cohort.**

1. UMAP embedding of cells derived from scRNA-seq data, including 18 ccRCC tissues. (B) Dot plot illustrating the expression patterns of marker genes for each cell type in scRNA-seq data. (C) Expression levels of AP-1 genes in ccRCC cells based on scRNA-seq data.

**Supplementary Figure 5**

**
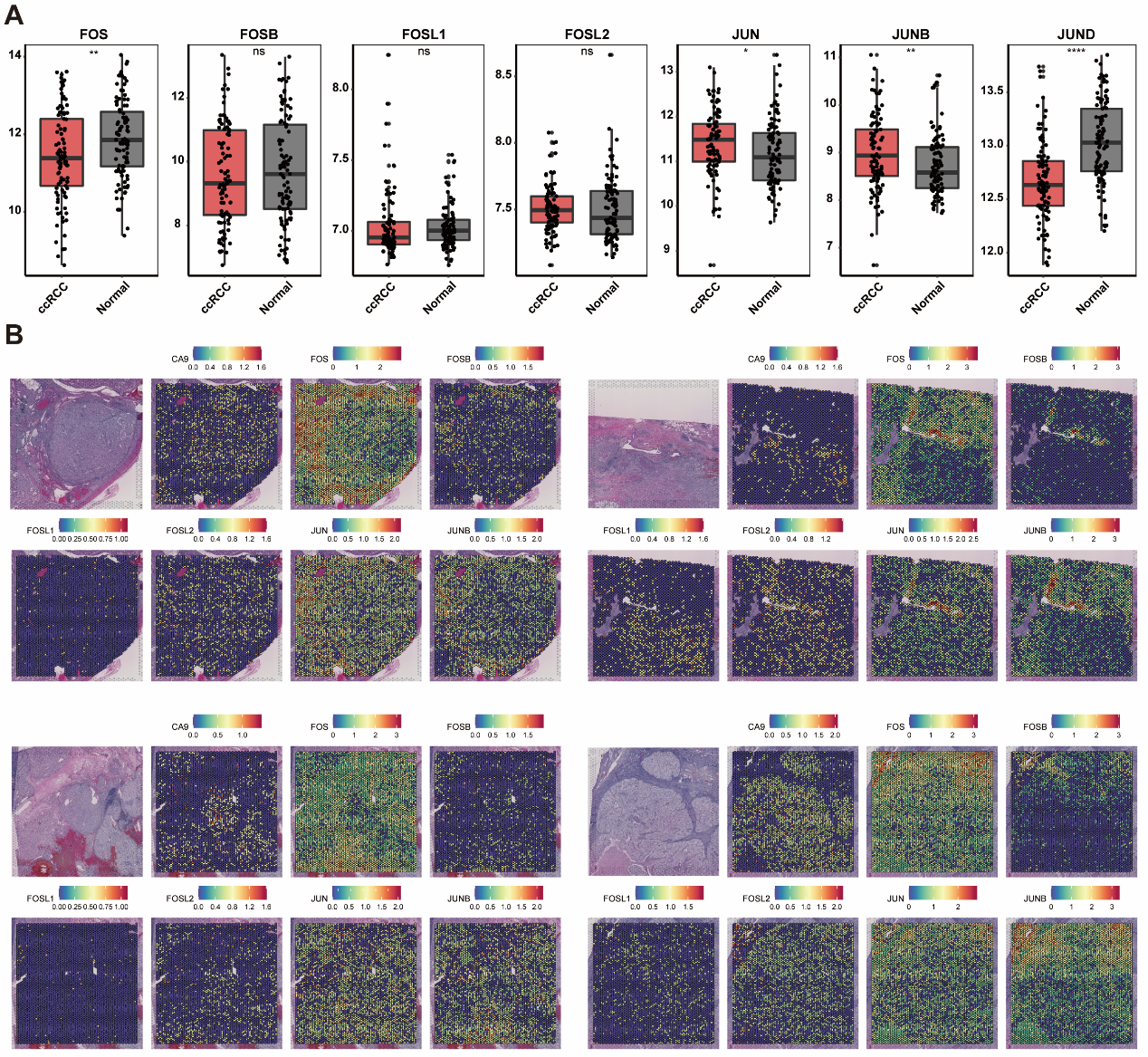
**

**Figure S5. Comparing FOS and JUN family gene expression between ccRCC and normal kidney samples and the spatial transcriptome expression of AP-1 (A)** Comparison of FOS and JUN family gene expression between ccRCC and normal kidney samples based on bulk-RNA validation cohort. The red represented ccRCC tumor samples, the gray represented normal kidney samples. (B) Spatial expression of AP-1 and CA9 based on stRNA-seq. CA9 is a specific marker of ccRCC cancer cells.

**Supplementary Figure 6**

**
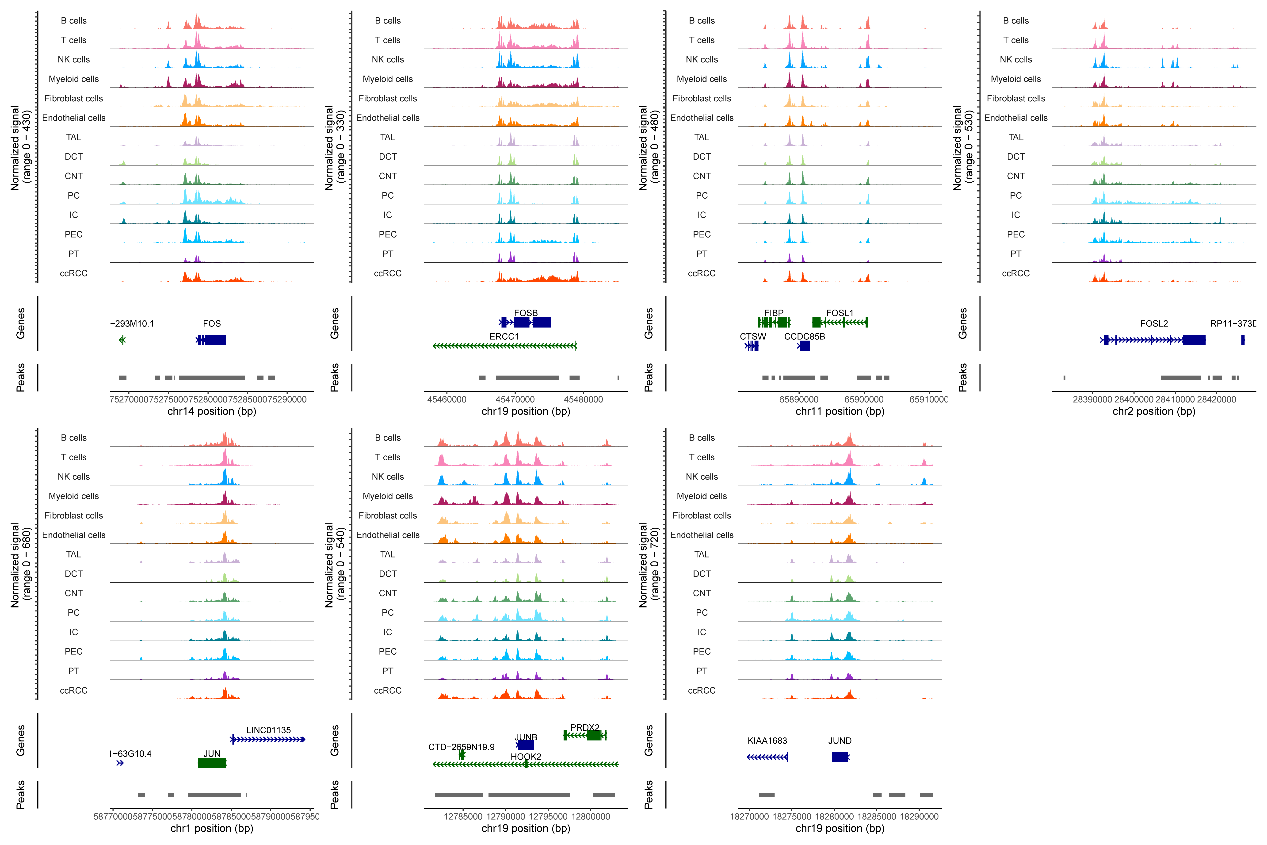
**

1. **Figure S6. Chromatin accessibility of AP-1 in scATAC-seq data.** cRCC, clear cell renal cell carcinoma; CNT, connecting tubule; DCT, distal convoluted tubule; IC, Intercalated cells; PC, principal cell; PEC, parietal epithelial cells; PT, proximal tubule; TAL, thick ascending limb.

**Supplementary Figure 7**

**
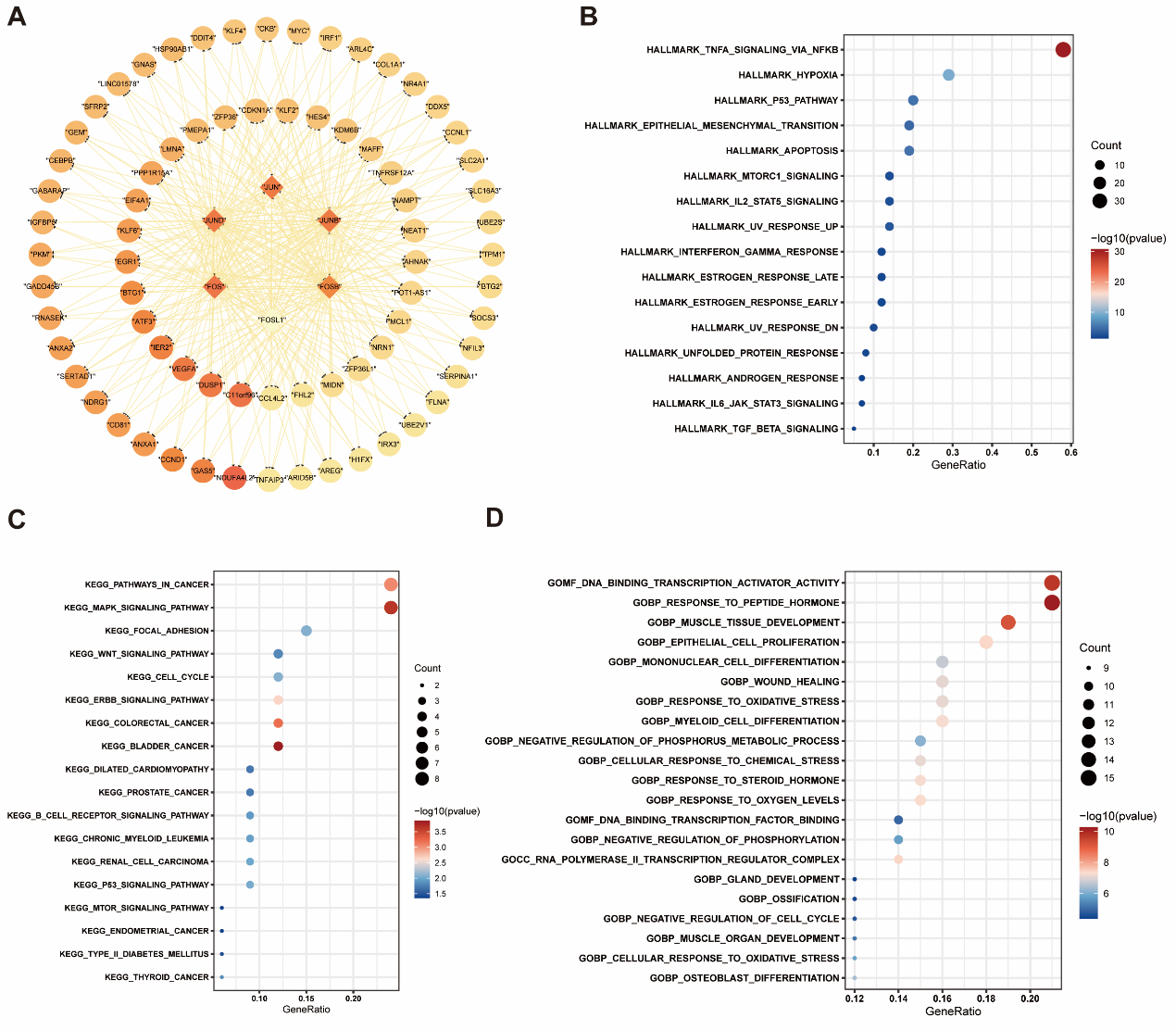
**

**Figure S7. Functional enrichment analysis based on AP-1 target genes**

(A) Identification of AP-1 target genes based on pySCENIC analysis. The inner layer of the circle represents co-targeting of more than 4 AP-1 family genes, while the outer layer of the circle represents co-targeting of less than 4 AP-1 family genes. (B) Functional enrichment analysis of AP-1 target-genes based on HALLMARK pathway gene sets. (C) Functional enrichment analysis of AP-1 target-genes based on KEGG pathway gene sets. (D) Functional enrichment analysis of AP-1 target-genes based on GO pathway gene sets.

**Supplementary Table 1**

**Table S1** Data source and Quality control in this study.

**Supplementary Table 2**

**Table S2** Differential motif activities in cancer cells vs. proximal tubule cells.

**Supplementary Table 3**

**Table S3** Target gene of AP-1.

**SUPPLEMENTARY METHODS**

**Data Acquisition**

The utilized data sources in this study are summarized in Table S1. For the discovery cohort, we employed three ccRCC scRNA-seq datasets^1^ and three normal kidney scRNA-seq datasets^2^. Additionally, we incorporated three ccRCC scATAC-seq datasets^1^ along with five kidney scATAC-seq datasets^3^. Spatial transcriptomics data of ccRCC were obtained from the Gene Expression Omnibus (GEO) database under accession number GSE175540^4^. The data utilized paraffin-embedded (FFPE) sections and consisted of four samples. Bulk-RNA data from the GDC-TCGA and GTEx databases were utilized in this study. The dataset included a total of 523 ccRCC samples and 100 normal kidney samples^5^ . In the validation cohort, we utilized a total of 18 paired ccRCC scRNA-seq and scATAC-seq datasets^6^. Bulk-RNA data of validation cohort was obtained from the GEO database under accession number GSE40435.

**Processing of scRNA-seq data**

The FASTQ raw data files of scRNA-seq were processed using Cell Ranger (version 6.1.2, 10x Genomics) with default parameters. The scRNA-seq data analyses were performed using the Seurat package in R software. Dimensionality reduction, clustering, and visualization were conducted using the SCTransform, RunPCA, and RunUMAP functions within the Seurat package. Cell quality control standards were organized in Table S1 and low-quality cells were filtered out. To address batch effects, the Harmony package (version 1.0) was applied for batch effects removal in the scRNA-seq analysis. Cell-type specific marker genes were identified using the FindAllMarkers function within the Seurat package, enabling manual annotation of each cell type in the scRNA-seq dataset.

**scATAC-seq data processing**

The FASTQ raw data files of scATAC-seq data were processed using Cell Ranger-atac-2.1.0 with default parameters. The scATAC-seq data was analyzed using the Signac pipeline (version 1.6.0) in R. Dimensionality reduction and clustering were performed using the RunT-FIDF, RunSVD, and RunUMAP functions within the Signac package. Cell quality control standards were organized in Table S1 and low-quality cells were filtered out. To address batch effects, the Harmony package (version 1.0) was applied for batch effects removal in the scATAC-seq analysis. Gene activity was determined by assessing the accessibility of chromatin peaks via the GeneActivity function in Signac, encompassing a region of the gene body and the upstream 2kb region of the transcription start site. Cell type annotations in the scATAC-seq data were determined based on the gene activities and chromatin accessibility of the corresponding marker genes. This information was used to assign cell types to individual cells in the scATAC-seq dataset.

**stRNA-seq data processing**

The stRNA data analysis was conducted using the Seurat package (version 4.2.0). Spot quality control standards were compiled and organized in Table S1, and spots with low quality were filtered out. The expression of relevant genes was visualized using the SpatialFeaturePlot function within the Seurat package.

**Bulk RNA-seq data analysis**

Bulk RNA-seq analyses of discovery cohort, including differential analysis and survival analysis of AP-1 genes, were conducted using the GEPIA platform (Gene Expression Profiling Interactive Analysis, http://gepia.cancer-pku.cn/)^7^. The differential analysis of AP-1 genes in validation cohort were conducted using R software.

**Motif analysis**

In the analysis of scATAC-seq data, we utilized chromVAR (v1.20.0) in R to evaluate TF binding accessibility profiles. This software calculates biased-corrected deviations, referred to as motif scores, which indicate the gain or loss of accessibility for each TF motif relative to the average cell profile. The motif position frequency matrices used in the analysis were obtained from the JASPAR2020 R package (v.0.99.10).

**Footprint analysis**

Transcription factor footprint analysis was performed using the Footprint function of the Signac R package. The reference data used for the analysis were obtained from the BSgenome.Hsapiens.UCSC.hg38 R package (v.1.4.4). These references provide the necessary genomic information for identifying transcription factor footprints in the scATAC-seq data.

### Transcription factor regulatory network analysis

The regulatory networks of transcription factors with target genes were defined using pySCENIC. Specifically, genes that were upregulated in ccRCC cancer cells were selected to identify the target genes of AP-1. The resulting TF regulatory network was visualized using Cytoscape software (v.3.10.1), allowing for a graphical representation of the interactions between transcription factors and their target genes.

**Functional enrichment analysis**

The functional enrichment analysis of the AP-1 target genes was conducted using the enricher function of the clusterProfiler package (version 4.2.2) in R. This analysis utilized gene sets from the HALLMARK, KEGG, and GO categories obtained from the Molecular Signatures Database (MSigDB). Significance was determined using an adjusted p-value threshold of less than 0.05, indicating significant differences in enriched functional terms associated with the AP-1 TF target genes.

**Statistical analysis**

All data analyses were performed using the R software (version 4.1.2). For the differential analysis of AP-1 expression and motif activity, the Wilcoxon test was utilized. A threshold of p < 0.05 was considered statistically significant for determining differential expression and motif activity of AP-1.

**Supplementary Information References**

1. Long Z, Sun C, Tang M, Wang Y, Ma J, Yu J, Wei J, Ma J, Wang B, Xie Q, et al. Single-cell multiomics analysis reveals regulatory programs in clear cell renal cell carcinoma. *Cell Discov*. Jul 19 2022;8(1):68. doi:10.1038/s41421-022-00415-0

2. Liao J, Yu Z, Chen Y, Bao M, Zou C, Zhang H, Liu D, Li T, Zhang Q, Li J, et al. Single-cell RNA sequencing of human kidney. *Sci Data*. Jan 2 2020;7(1):4. doi:10.1038/s41597-019-0351-8

3. Muto Y, Wilson PC, Ledru N, Wu H, Dimke H, Waikar SS, Humphreys BD. Single cell transcriptional and chromatin accessibility profiling redefine cellular heterogeneity in the adult human kidney. *Nat Commun*. Apr 13 2021;12(1):2190. doi:10.1038/s41467-021-22368-w

4. Meylan M, Petitprez F, Becht E, Bougouin A, Pupier G, Calvez A, Giglioli I, Verkarre V, Lacroix G, Verneau J, et al. Tertiary lymphoid structures generate and propagate anti-tumor antibody-producing plasma cells in renal cell cancer. *Immunity*. Mar 8 2022;55(3):527-541 e5. doi:10.1016/j.immuni.2022.02.001

5. Cancer Genome Atlas Research N. Comprehensive molecular characterization of clear cell renal cell carcinoma. *Nature*. Jul 4 2013;499(7456):43-9. doi:10.1038/nature12222

6. Yu Z, Lv Y, Su C, Lu W, Zhang R, Li J, Guo B, Yan H, Liu D, Yang Z, et al. Integrative Single-Cell Analysis Reveals Transcriptional and Epigenetic Regulatory Features of Clear Cell Renal Cell Carcinoma. *Cancer Res*. Mar 2 2023;83(5):700-719. doi:10.1158/0008-5472.CAN-22-2224

7. Tang Z, Li C, Kang B, Gao G, Li C, Zhang Z. GEPIA: a web server for cancer and normal gene expression profiling and interactive analyses. *Nucleic Acids Res*. Jul 3 2017;45(W1):W98-W102. doi:10.1093/nar/gkx247
